# Machine learning models for delineating marine microbial taxa

**DOI:** 10.1093/nargab/lqaf090

**Published:** 2025-06-19

**Authors:** Stilianos Louca

**Affiliations:** Department of Biology, University of Oregon, Eugene, OR 97403, United States; Institute of Ecology and Evolution, University of Oregon, Eugene, OR 97403, United States

## Abstract

The relationship between gene content differences and microbial taxonomic divergence remains poorly understood, and algorithms for delineating novel microbial taxa above genus level based on multiple genome similarity metrics are lacking. Addressing these gaps is important for macroevolutionary theory, biodiversity assessments, and discovery of novel taxa in metagenomes. Here, I develop machine learning classifier models, based on multiple genome similarity metrics, to determine whether any two marine bacterial and archaeal (prokaryotic) metagenome-assembled genomes (MAGs) belong to the same taxon, from the genus up to the phylum levels. Metrics include average amino acid and nucleotide identities, and fractions of shared genes within various categories, applied to 14 390 previously published non-redundant MAGs. At all taxonomic levels, the balanced accuracy (average of the true-positive and true-negative rate) of classifiers exceeded 92%, suggesting that simple genome similarity metrics serve as good taxon differentiators. Predictor selection and sensitivity analyses revealed gene categories, e.g. those involved in metabolism of cofactors and vitamins, particularly correlated to taxon divergence. Predicted taxon delineations were further used to *de novo* enumerate marine prokaryotic taxa. Statistical analyses of those enumerations suggest that over half of extant marine prokaryotic phyla, classes, and orders have already been recovered by genome-resolved metagenomic surveys.

## Introduction

Recent advances in genome-resolved metagenomics provide unprecedented opportunities for discovering and classifying novel bacterial and archaeal (henceforth “prokaryotic”) taxa and for estimating global prokaryotic taxonomic diversity [1–3]. However, developing methods for classifying prokaryotes into higher level taxonomic units based on genomic content remains a challenge [4]. One reason is that current taxonomic classifications are strongly shaped by historical conventions that evolved prior to the wide availability of genomic data, and whose quantitative relationship to genomic metrics of divergence is far from obvious. Nearly all past attempts to delineate prokaryotic taxa based on genomic comparisons are based on average amino acid identity (AAI), average nucleotide identity (ANI), and/or comparisons of a small set of universal phylogenetic marker genes [5–10]. Some studies have developed criteria to delineate genera based on the fraction of shared genes or, more broadly, genome alignment fractions [11, 12]. Their applicability, however, to higher taxa remains unexplored, and it is unclear how much more practically accessible information gene content carries relative to molecular similarity metrics such as AAI or ANI. Similarly, the relative involvement of different types of genes in higher level taxonomic divergence remains unknown. For example, it is unknown whether the presence/absence of genes involved in metabolism, a common axis of ecological differentiation in prokaryotes, carries more phylogenetic signal and discriminatory power for taxonomic classification than other genes. Further, while the 16S rRNA gene has historically been widely used for prokaryotic systematics [13], it is usually poorly covered in metagenome-assembled genomes (MAGs) due to assembly difficulties [14], necessitating the development of taxonomic classification techniques that do not rely on 16S. Exploring genome-based techniques for delineating higher prokaryotic taxa, and determining the relative informational value of different types of genes for taxonomic delineations, are worthwhile goals in and of themselves. Indeed, ifmsuch work would yield insight into the interaction between deep clade divergence and broad-scale ecological niche differentiation [15, 16], it would help improve the accuracy of prokaryotic taxonomies and phylogenies [3], and would even enable more accurate estimations of global extant prokaryotic diversity.

Here, to address the above gaps, I analyzed the genomic similarities and taxonomic relationships between thousands of publicly available marine prokaryotic MAGs from >100 surveys across the world’s oceans. I focus on the marine microbiome due to (i) its importance for Earth’s biogeochemical cycles [17], (ii) the large number of available marine metagenomic surveys, (iii) the relatively well mixed state of the ocean and thus geographically wide detectability of its inhabiting prokaryotes [18–20], and (iv) the lack of prokaryotic taxonomic diversity estimates for the ocean. At each taxonomic level (phylum down to genus), I trained a neural network model [21] to predict whether any two genomes are in the same taxon or not, based on a set of candidate genome similarity metrics, including AAI, ANI, and fractions of shared genes (FSG) restricted to various Kyoto Encyclopedia of Genes and Genomes (KEGG) gene categories [22]. Through predictor selection and sensitivity analysis, I identified gene categories of particular informative value for delineating marine prokaryotic taxa. I then used the pairwise classifications between MAGs as inputs to clustering algorithms to reconstruct taxonomic relationships *de novo* within the dataset, including taxa not currently defined in taxonomic reference databases [10, 23]. Lastly, using taxon accumulation curves and statistical richness estimators, I estimate the global number of marine prokaryotic taxa and discuss the potential for future surveys to keep uncovering novel prokaryotic diversity in the ocean.

## Materials and methods

This section provides technical details on the materials and methods used. A more readable overview is interspersed throughout the Results and discussion.

### MAGs

MAGs from 106 metagenomic surveys were downloaded from either the NCBI GenBank or other locations provided by published surveys. Only surveys in which all MAGs were obtained from the marine environment or in which the environment was specified for each MAG were considered. For the latter, only MAGs reportedly from marine environments were kept. For efficiency in MAG collection, I focused on surveys with at least 50 MAGs. Surveys focusing on a single clade (e.g. *Thaumarchaeota*) or trait (e.g. only methanogens) were omitted. An overview of included surveys, including accession numbers and publication references, is provided in Supplementary Data S1. With the exception of one study (‘Genomes from Earth’s Microbiomes’ or GEM; [24]), all other surveys focused on the marine environment. MAG qualities were determined based on the presence or absence of various universal single-copy genes using checkM2 v1.0.0 [25]. MAGs with completeness <80% or contamination >5% were omitted; thus 26 466 MAGs were kept for analysis. An overview of completeness and contamination levels of those MAGs which were kept is shown in Supplementary Fig. S1; details are available in Supplementary Data S2.

Taxonomic identities of MAGs were determined using the Genome Taxonomy Database (GTDB)-Tk v2.3.2 workflow classify_wf [23], except for the GEM MAGs for which taxonomic identities were already provided by the original study. An overview of named prokaryotic phyla and classes is shown in Supplementary Fig. S2. Protein-coding genes were detected in MAGs using prodigal v2.6.3, and were subsequently annotated (matched to KEGG orthologs) using the KOfam Hidden Markov Model database [26] (release 2020-04-02) and hmmsearch v3.3.2 [27]. ANIs and AAIs between MAGs were computed using mash v2.3 [28], with sketch size 5000 and otherwise default options. MAGs were subsequently binned into species-level genome bins at an ANI cut-off of 95%, using a hierarchical clustering approach described previously [29]. All software mentioned above were used with default options unless specified otherwise. A total of 14 390 species genome bins were thus obtained. A list of MAGs in each bin is available as Supplementary Data S3. From each bin, a single representative MAG was kept, chosen to be the MAG with the highest completeness. Unless otherwise mentioned, all downstream analyses were performed using these representative MAGs, to avoid representational imbalances between species and avoid redundancies; the original set of MAGs is referred to as “raw MAGs”.

FSGs between any two MAGs were assessed separately within each considered category of genes, with categories defined according to the KEGG hierarchy, levels A and B [30]. For example, I computed FSGs within all of KEGG, as well as within the KEGG level A category “metabolism”, the KEGG level A category “environmental information processing”, and so on. A total of 26 gene categories deemed relevant to prokaryotes were considered (overview in Supplementary Table S1). For any given gene category, and for any two MAGs, FSG was computed as the estimated ratio between the number of shared genes and the number of genes present in any of the two represented organisms, while accounting for MAG completeness. Note that this ratio is also known as the Jaccard similarity index [31, 32]. Specifically, let *N*_1_, *N*_2_, *N*_12_ be the number of genes found in the first MAG, the second MAG, and in both MAGs, respectively, and let *C*_1_ and *C*_2_ be the completeness of the first and second MAG, respectively (estimated using checkM2). Then FSG was computed as:


(1) \begin{eqnarray*}
\text{FSG} = \frac{\hat{N}_{12}}{\hat{N}_1+\hat{N}_2-\hat{N}_{12}},
\end{eqnarray*}

where:


(2) \begin{eqnarray*}
\hat{N}_1:=\frac{N_1}{C_1},\quad \hat{N}_2:=\frac{N_2}{C_2},\quad \hat{N}_{12}:=\frac{N_{12}}{C_1C_2}.
\end{eqnarray*}

### Predicting taxonomic delineations

Henceforth, at any given taxonomic level (e.g. genus, family, etc.), any two MAGs are referred to as “ingroup” if they belong to the same taxon, and as “outgroup” otherwise. For example, at genus level, two MAGs are referred to as ingroup if they are congeneric. Note that, since not all MAGs could be identified at all taxonomic levels using GTDB-Tk, the ingroup/outgroup status was not *apriori* known for all MAG pairs. For any given taxonomic level, a binary classification model was constructed that predicts for any two MAGs whether they are an ingroup or outgroup. Hence, a single data point for the model represents a specific pair of MAGs with known ingroup/outgroup status.

As model class, I considered neural networks, also known as multilayer perceptrons, implemented in the python package sklearn v1.4.0, module neural_network [33]. As potential predictor variables, I considered the AAI, ANI, and the FSGs specific to the 26 gene categories described above. Missing AAI and ANI values, i.e. which could not be computed due to the low relatedness between some MAGs, were replaced with a value of 0. The optimal subset of predictors, as well as the optimal structure of the neural network (aka “hyperparameters”), including the number and sizes of hidden layers, the activation function, and the L2 regularization strength (“alpha”), were selected by maximizing the average balanced accuracy achieved during 10-fold cross-validation. The balanced accuracy is defined as 0.5 × (*P* + *N*), where *P* is the true positive rate and *N* is the true negative rate of the classifier. This metric was chosen because it accounts for imbalances in the input data, i.e. strong differences between the numbers of ingroup and outgroup MAG pairs, while still being easily interpretable and easy to connect to the more fundamental true-positive and true-negative rates. For any given predictor subset and choice of hyperparameters, the model was fitted by minimizing the log-loss function using the LBFGS optimization algorithm. The space of possible predictor subsets and hyperparameters was explored iteratively, by nesting the hyperparameter selection into the predictor subset selection, as follows. For any given candidate predictor subset, I examined all hyperparameter choices resulting from a combination of allowed alpha values (10^−5^, 10^−4^, 10^−3^, 0.01, 0.1, 1, 10), activation functions (identity, logistic, tanh), hidden layer counts (1 or 2), and various hidden layer sizes that depended on the size of the predictor subset. This grid search was performed using the sklearn function model_selection.GridSearchCV. The score for the particular predictor subset was thus the maximum score (balanced accuracy) achieved by any hyperparameter choice via 10-fold cross-validation using that predictor subset. Predictor subsets were explored non-exhaustively using a sequential bidirectional search method known as “plus *l* minus *r*” [34, 35], with *l* = 1 and *r* = 1. The search was halted once the balanced accuracy increased no more than 0.5%.

Data subsets for training, validation, and testing were constructed as follows. First, a random subset of 2 000 000 MAG pairs was selected as “input data” for computational efficiency. This was necessary to reduce computational requirements to a manageable level; tests using only 1 000 000 MAG pairs revealed no noteworthy differences, suggesting that this data size was sufficient for our purposes. Next, a test set was reserved by randomly choosing ∼10% of the input data. Next, among the remaining data (“non-test data”), 10 pairs of training–validation sets were chosen, independently of each other. Each validation set comprised ∼10% of the non-test data, chosen randomly. Each corresponding training set was constructed from the remaining non-test data, by balancing the number of ingroup/outgroup datapoints and constraining their total count to <20 000 via rarefaction for computational efficiency. Upon hyperparameter and predictor selection, the model was re-fitted on the non-test data after balancing via rarefaction, and its accuracy was evaluated on the test set. Finally, in preparation for downstream analyses, the model was re-fitted using the full input data (after rarefaction for balancing); these classifiers are henceforth referred to as “final”. The numbers of training/validation/test datapoints used at each taxonomic level are shown in Supplementary Table S2. Hyperparameters selected at each taxonomic level are shown in Supplementary Table S3. Accuracies achieved by classifiers on the test sets are shown in Table 1. Accuracies achieved using only FSGs as possible predictors are listed in Supplementary Table S4.

**Table 1.** Classifier performances (MAGs): overview of achieved classification accuracies (true positive rate, true negative rate, balanced accuracy) and similarity metrics selected as predictors, at each taxonomic level. For the unique contribution of each predictor to the classifier's balanced accuracy see Supplementary Table S5

Taxonomic level	True positive	True negative	Balanced accuracy	Predictors
Phylum	0.914	0.939	0.926	AAI,
				FSG KEGG,
				FSG KEGG B environmental adaptation,
				FSG KEGG B amino acid metabolism
Class	0.957	0.955	0.956	AAI,
				FSG KEGG B environmental adaptation,
				FSG KEGG B amino acid metabolism
Order	0.977	0.952	0.964	AAI,
				FSG KEGG
Family	0.996	0.983	0.989	AAI,
				FSG KEGG B xenobiotics biodegradation and metabolism
Genus	1	0.995	0.998	AAI,
				FSG KEGG B replication and repair

### Sensitivity analysis

To examine the importance of individual genome similarity metrics as predictors, I followed two alternative approaches. In the first approach (“forward sensitivities”), I repeated the classifier selection at each taxonomic level and for each genome similarity metric, while forcing the classifier to be based only on that single metric as a predictor. The hyperparameters of each such classifier were re-selected from scratch, as described earlier. To account for biases stemming from differences in the number of genes between gene categories, I randomly subsampled the genes in each category to the maximum possible common number, while only considering gene categories with at least 100 genes; thus, 22 gene categories were considered and subsampled to 115 genes. The true-positive rate, true-negative rate, and balanced accuracy of the obtained classifiers were estimated as described earlier, and yielded insight into the predictive power that could be achieved from any given predictor alone (Fig. 3; Supplementary Figs S3 and S4). For comparison, I repeated this analysis using FSGs based on non-rarefied gene sets (Supplementary Fig. S5), although I stress that the obtained balanced accuracies should not be compared between gene categories to assess the phylogenetic signal of individual genes in various gene categories. Note that when used for binary classification, these single-predictor classifiers are equivalent to simple threshold-based classifiers (thresholds given in Supplementary Data S8). If, however, these classifiers were to be used for probabilistic predictions (e.g. as described below), they would no longer be equivalent to a simple threshold-based classifier, and the internal neural structure and hyperparameters would influence the probabilities generated.

**Figure 1.**
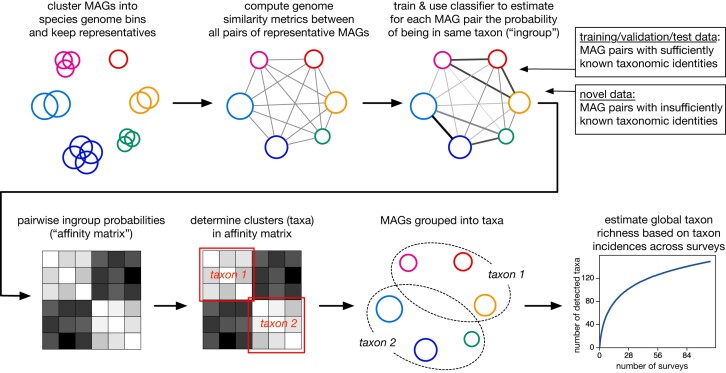
Workflow overview. Overview of the main steps followed for classifying MAG pairs as “ingroup” (i.e. in the same taxon) or “outgroup” (i.e. in a different taxon), grouping MAGs into taxa *denovo*, and estimating global taxon richness, separately for each taxonomic level.

**Figure 2.**
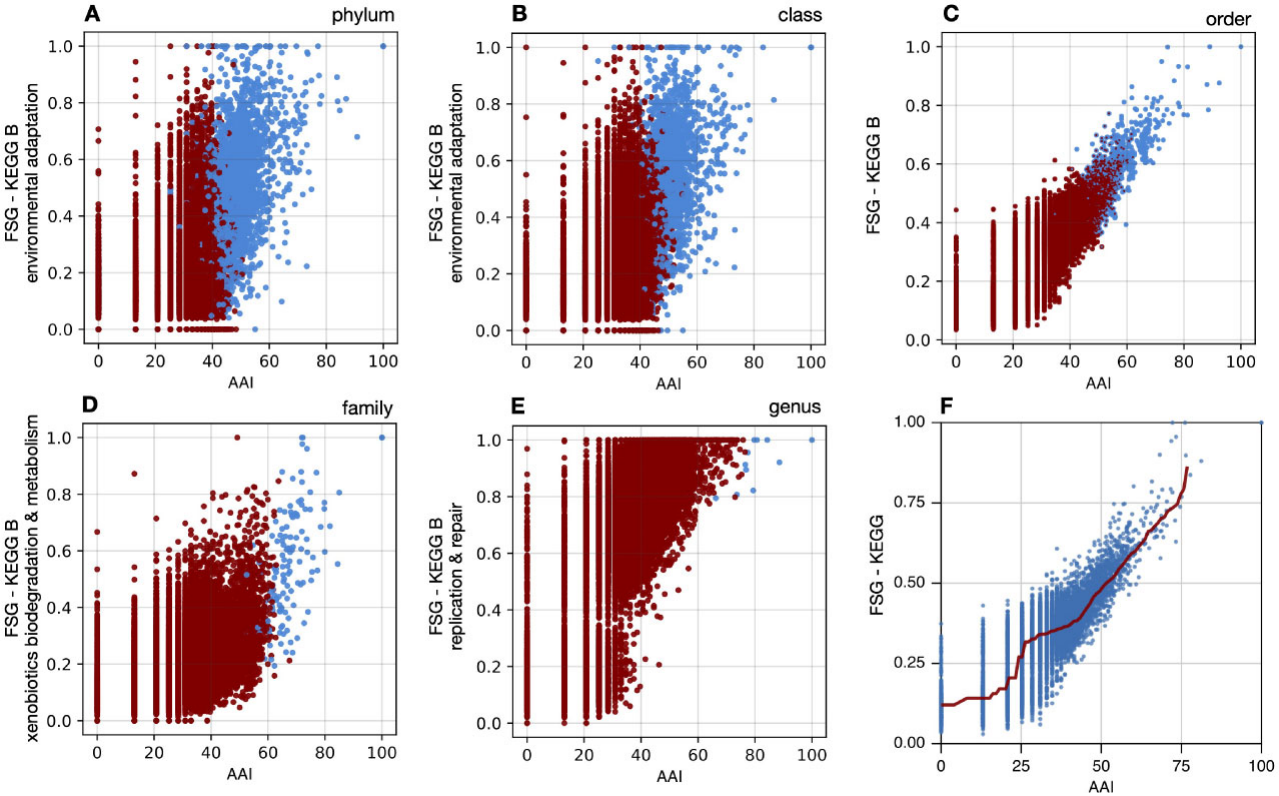
Top selected predictors. (**A–E**) Each plot shows the two predictors with the highest unique contributions to the classifier at a specific taxonomic level (Supplementary Table S5), evaluated between 20 000 randomly chosen MAG pairs. Each point represents a distinct MAG pair, and is colored according to whether the two MAGs are known to be in the same taxon (“ingroup”, blue points) or known to be in a different taxon (“outgroup”, red points). For example, A shows pairwise AAIs and FSGs (within the KEGG B category “environmental adaptation”) between MAGs, the two most important predictors of the selected classifier at phylum level. (**F**) Pairwise AAIs and FSGs (all KEGG) between MAGs. The red curve shows the rolling median FSG value, with a rolling window spanning 10% of the total AAI range. For a similar plot that also shows classifier predictions, see Supplementary Fig. S7.

**Figure 3.**
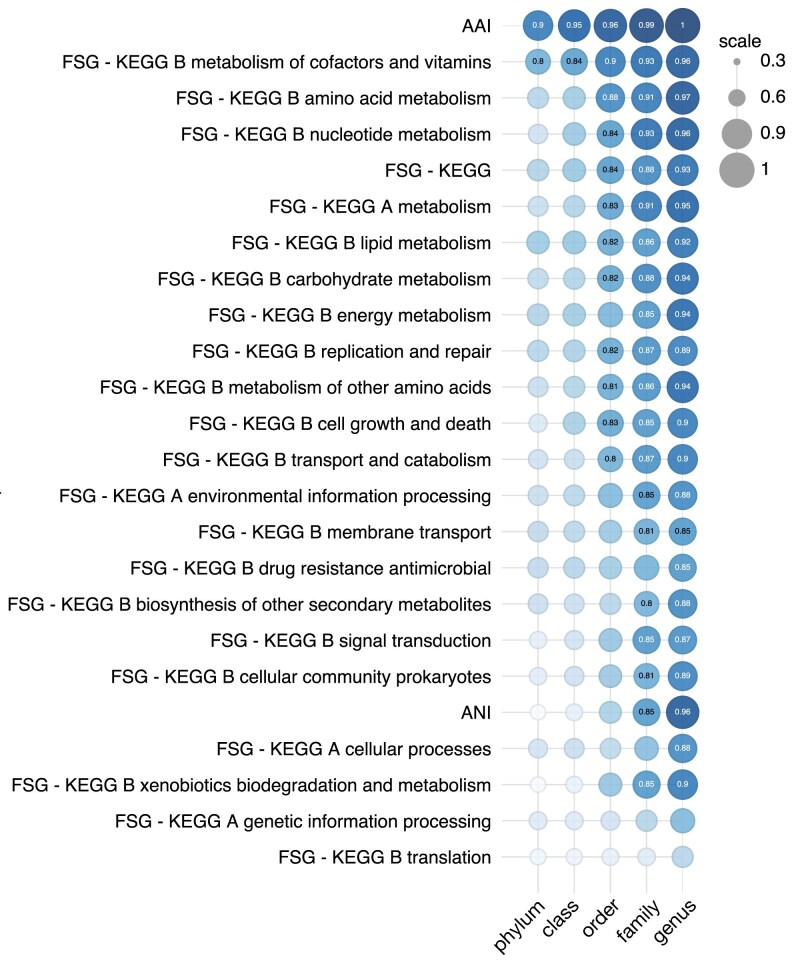
Balanced accuracies achieved by individual similarity metrics (rarefied gene sets). Balanced classification accuracies achieved by models based on individual pairwise similarity metrics between MAG, including AAI (average amino acid identity), ANI (average nucleotide identity), and FSGs (fractions of shared genes, restricted to various KEGG categories), separately for each taxonomic level. Larger and darker circles correspond to higher accuracies. Balanced accuracies ≥0.8 are written inside the circles. Metrics are listed in order of decreasing balanced accuracy (averaged over all taxonomic levels). Prior to computing FSGs, KEGG categories were rarefied to the same number of genes (115) in order to avoid size-driven biases. For analogous figures showing true-positive and true-negative rates, see Supplementary Figs S3 and S4, respectively. For detailed values, see Supplementary Data S7. For balanced accuracies achieved with FSGs within non-rarefied gene categories, see Supplementary Fig. S5. For a comparable figure for RefSeq genomes, instead of MAGs, and also considering 16S similarities as predictor, see Supplementary Fig. S9.

In the second approach (“backward sensitivities”), I started with the original set of predictors selected at a specific taxonomic level, and removed one predictor at a time, each time re-selecting hyperparameters, training the classifier, and assessing its balanced accuracy. The reduction in the balanced accuracy, when compared with the original classifier, was taken as a proxy for the contribution of a given predictor to the original classifier (e.g. Fig. 2). Detailed results are listed in Supplementary Table S5.

### Enumerating tax*a* *de novo*

In order to estimate the total number of taxa and the member MAGs in each taxon, I used various clustering algorithms, whose sole input is an “affinity matrix” that lists pairwise ingroup probabilities between MAGs. For MAG pairs with unknown ingroup/outgroup status, probabilities were predicted with the final classifiers using the method predict_proba. For MAG pairs with known ingroup/outgroup status (i.e. whose taxonomic identities could be sufficiently resolved with GTDB-tk), probabilities were only predicted during the cross-validation tests (described below) but set to 1 or 0 as appropriate for the final taxon enumerations; the last step was performed because the primary goal at that point was to estimate global prokaryotic richness using as much information as possible. The following clustering algorithms were considered: Affinity Propagation [36], DBSCAN [37], HDBSCAN [38], and two custom greedy clustering algorithms (GG and GGR) described in Supplementary S1. For the first three algorithms, the implementations provided in the python package sklearn, module cluster, were used with default parameters. GG and GGR were implemented from scratch in python (code in Supplementary Data S6). GG and GGR depend on a numeric parameter, σ, which can range between 0 and 1; various values of σ were henceforth considered (0.5, 0.6, 0.7, 0.8, 0.9, 0.95, 0.99, and 0.995). All algorithms take as input a matrix of pairwise affinities, which in our case represented the estimated ingroup probabilities, with values ranging between 0 and 1.

The accuracy of each clustering algorithm was assessed using cross-validation, as follows. Random subsets of MAGs with known taxonomic identities were used as input to the final classifier, then pairwise classification probabilities were predicted *de novo* for all MAGs in the subset (even for MAGs with highly resolved taxonomic identities), the number of taxa were predicted by the clustering algorithm, and finally that number was compared with the true number of input taxa. Various MAG subset sizes were examined, and the accuracy of each clustering algorithm was measured in terms of the fraction of variance in taxon counts explained by the clustering algorithm’s predictions (*R*^2^). At each taxonomic level, the clustering algorithm with the highest *R*^2^ was used for subsequent analyses. This optimal algorithm was then used to estimate the total number of taxa (clusters) in the entire MAG dataset. An overview of selected clustering algorithms, associated *R*^2^, and number of taxa predicted for this dataset are given in Supplementary Table S6. A visualization of test results is shown in Supplementary Fig. S6. An overview of the above workflow is shown in Fig. 1.

To assess the effect of incorporating known taxonomic relationships into the affinity matrix (i.e. fixing the probabilities for MAG pairs with *a priori* known ingroup/outgroup status), I repeated the above richness estimates for the present MAG dataset after predicting from scratch all ingroup/outgroup statuses regardless of whether these were known or not. The newly obtained estimates were nearly identical to those obtained earlier, differing by <5% at all taxonomic levels (Supplementary Table S6).

### Global taxon richness estimates

**Figure 4.**
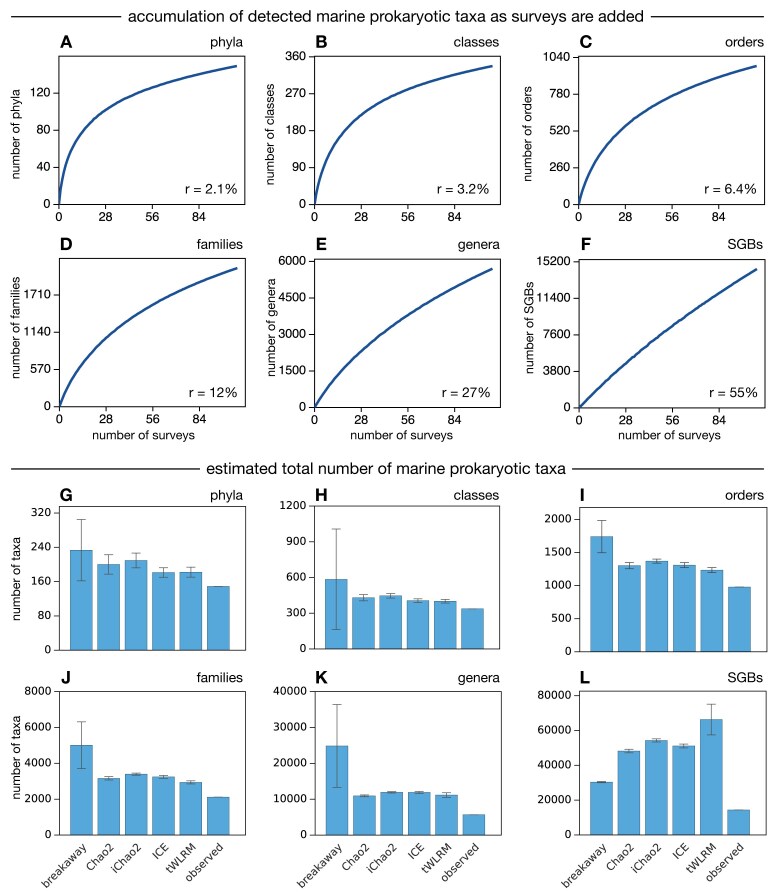
Taxon accumulation curves over MAGs. (**A**) Accumulation curve of marine prokaryotic phyla as a function of the number of surveys. Each point in the curve shows the expected number of phyla in a specific number of randomly chosen MAG surveys. The number of phyla was estimated *de novo* based on detected MAGs and using a clustering algorithm, as described in the text. The ratio of the final (i.e. right-most) slope over the initial (i.e. left-most) slope, denoted *r*, is shown in the figure; this slope is the fraction of phyla detected in a new MAG study that are expected to be novel. (**B–E**) Similar to (A), but for classes, orders, families, and genera. (**F**) Accumulation curve of species genome bins as the number of surveys increases. For accumulation curves as a function of the number of raw MAGs, see Supplementary Fig. S8. (**G–L**) Estimated global number of marine prokaryotic taxa or species genome bins, based on incidence frequencies across MAG surveys and using various estimation algorithms. The last bar in each plot shows the number of taxa observed in the present data, for comparison. Whiskers show standard errors, estimated from the models; most standard errors are likely to be an underestimate of the true uncertainty, hence the variability between models is a better reflection of uncertainty.

To estimate the total number of extant marine prokaryotic taxa, I proceeded as follows. Based on the taxon–MAG associations predicted earlier, I computed the incidence frequency counts *Q*_1_, *Q*_2_, … of taxa across surveys, where each *Q*_*n*_ is the number of taxa detected in exactly *n* surveys. I then applied previously published statistical estimation methods based on the *Q*_1_, *Q*_2_, …. To assess the robustness of my estimations, I considered several alternative methods, based on different frequency models and relying on different assumptions: the Chao2 richness estimator [39], based on the frequency counts *Q*_1_–*Q*_2_; the improved-Chao2 (“iChao2”) richness estimator [40], based on the frequency counts *Q*_1_–*Q*_4_; the incidence coverage-based estimator (ICE) [39], based on the frequency counts *Q*_1_–*Q*_10_; the transformed weighted linear regression model (tWLRM), which uses a linear regression model for the ratios of consecutive log-transformed frequency counts to predict *Q*_0_ [41, 42]; and the breakaway estimator [43], based on a non-linear regression model for the ratios of consecutive frequency counts. Each of the above estimators accounts for heterogeneities in detection frequencies among taxa (i.e. the presence of rare and frequent taxa), and breakaway is particularly optimized for dealing with high fractions of undiscovered diversity. Observe that these estimators rely on the low-frequency counts *Q*_1_, *Q*_2_, etc. Indeed, widely distributed taxa, i.e. which are almost certain to be detected, contain only little information about undetected taxa, while rarely detected taxa carry the most information about undetected taxa [44]. An overview of richness estimates obtained by each estimator and at each taxonomic level is shown in Fig. 4G–L.

To visually asses the likely global number of undetected marine prokaryotic taxa, I also computed taxon accumulation curves (Fig. 4A–F). These curves display the expected number of taxa detected in any given number of MAG surveys, when surveys are randomly chosen from the full dataset. The value of each accumulation curve at any given number of surveys *n* was computed by randomly choosing *n* surveys, computing the number of taxa detected in those *n* surveys, repeating this process 10 000 times, and averaging over all repeats.

## Results and discussion

### A diverse collection of MAGs

A total of 26 466 marine prokaryotic MAGs were obtained from 106 previous ocean microbiome surveys worldwide, including the coastal and deep ocean, estuaries, lagoons, marine sediments, hydrothermal vents, seafloor aquifers, tidal flats, peritidal stromatolites, antarctic ice shelfs, corals, sea sponges, clams, and other marine eukaryotic hosts (overview and accession numbers in Supplementary Data S1). All considered MAGs were estimated to be at least 80% complete and exhibit no more than 5% contamination, based on several universal single-copy marker genes (Supplementary Fig. S1). MAGs covered 89 named phyla and an additional 60 candidate phyla as defined by the GTDB [10]. To avoid imbalances in species representation and reduce redundancy, I binned MAGs into 14 390 species-level genome bins based on the commonly used ANI threshold of 95%, and thereafter only considered one representative MAG per bin [8, 45, 46]. Thus, ANIs, AAIs, and FSGs were computed for a total of 207 072 100 MAG pairs.

### Neural network classifiers for delineating taxa

To examine the predictive power of pairwise genome similarity metrics for delineating marine prokaryotic taxa, I used these metrics as a pool of possible predictor variables to construct a neural network model [21], which classifies any two MAGs as either being in the same taxon (henceforth “ingroup” or “positive”) or in distinct taxa (henceforth “outgroup” or “negative”). A separate classifier was constructed at each taxonomic level, i.e. at phylum level, class level, and so on down to genus level. Each pair of MAGs constituted one data point, with the training, validation, and test data comprising MAG pairs that exhibited sufficiently precise taxonomic identifications such that their delineation status was *a priori* known. The classifiers thus predicted whether any given MAG pair is in the same taxon (ingroup) or, more generally, yielded a probability of being in the same taxon. Note that this approach differs from the conventional practice of delineating higher prokaryotic taxa based on phylogenetic distance and experimentally determined traits [13]. The performance of each classifier was evaluated in terms of its balanced accuracy, i.e. the average of the true-positive rate (sensitivity) and the true-negative rate (specificity). The optimal subset of genome similarity metrics used as predictors by a model, as well as the optimal hyperparameters (e.g. hidden layer sizes and activation function), were chosen using an iterative search based on cross-validation, following common practice in the field of machine learning (47, 48; see the Materials and methods for details).

At all taxonomic levels, classifiers achieved a high balanced accuracy when tested on an independent dataset, ranging from 0.926 at phylum level up to 0.998 at genus level (Table 1; Supplementary Fig. S7). The true-positive rate ranged from 0.914 at the phylum level up to 1.0 at the genus level, while the true-negative rate ranged from 0.939 at the phylum level up to 0.995 at the genus level. This shows that even higher taxa can be accurately delineated by neural network models using genome similarity metrics as predictors. It is worth pointing out that classifier performance was greatest at genus level in terms of both the true-positive rate and the true-negative rate, despite the fact that fewer datapoints were available for training this classifier (overview in Supplementary Table S2).

At all taxonomic levels, erroneous classifications mostly occurred for marginal cases, i.e. for which predictor values were close to the boundary separating the two outcomes (Supplementary Fig. S7). In fact, a quick look at individual cases (Supplementary Data S9) reveals that erroneous classifications at a given taxonomic level are typically correct when interpreted at the next taxonomic level, i.e. one level below or above in the case of a false-positive or false-negative prediction, respectively. For example, MAGs GCA_902529805.1 and GCA_902597545.1 [49] were erroneously classified as belonging to the same order, despite being identified as *Cytophagales* and *Flavobacteriales*; however, they do belong to the same class of *Bacteroidia*. One explanation may be that misclassifications stem mostly from a noisy relationship between our partly arbitrary and noisy taxonomic system on the one hand, and the underlying biology on the other hand.

The number of selected predictors ranged from two to four depending on taxonomic level (Table 1). In all cases, AAI was one of the selected predictors (Fig. 2), and was always the predictor with the highest unique contribution, measured in terms of the reduction of balanced classification accuracy when omitted as a predictor (Supplementary Table S5). ANI was never selected as a predictor, suggesting that its usefulness for delineating taxa is mostly restricted to the species level [8]. I emphasize that predictor contributions to classifier accuracy need not be additive. For example, predictors can correlate and thus exhibit redundancies relative to each other. For example, even when omitting AAI from models, the balanced accuracy never decreased by more than 0.1 (Supplementary Table S5), which means that the remaining predictors in each model still had substantial predictive power. Moreover, when AAI and ANI were excluded from the predictor selection process, i.e. considering only FSGs as possible predictors, the selected classifiers were almost as accurate as those achieved when AAI and ANI were considered (balanced accuracies between 0.897 and 0.99, Supplementary Table S4). This suggests that information on taxonomic affiliation carried by AAI is largely shared with various FSGs. In fact, in all cases, a larger number of FSGs was selected when AAI/ANI were excluded compared with when AAI/ANI were allowed, indicating that the information lost by excluding AAI/ANI was partly replaced by including more FSGs.

To further examine the predictive power of each genome similarity metric on its own, I also constructed a classifier using one metric at a time as predictor, and assessed the classifier’s balanced classification accuracy. This balanced accuracy, achieved by a classifier based on a given single metric, serves as an indication of that metric’s informative value and ultimately its phylogenetic signal. It is important to note that various gene categories (e.g. KEGG A “metabolism” and KEGG A “cellular processes”) can comprise vastly different numbers of genes, which can affect the stochasticity of the derived FSGs and their ability to carry a phylogenetic signal. Thus, even if individual genes were to all carry a similar phylogenetic signal, FSGs within larger gene categories would tend to express this signal more, essentially due to the law of large numbers. Indeed, in preliminary tests, I observed that FSGs within larger gene categories tended to achieve higher classifier accuracies, thus making it difficult to draw conclusions about the phylogenetic signal of individual genes in each category. To avoid these biases, I randomly subsampled (rarefied) all gene categories to the largest possible common number of genes, while considering only categories with at least 100 genes. An overview of achieved balanced accuracies is shown in Fig. 3. At all taxonomic levels, AAI stood out as the single metric with the highest balanced classifier accuracy, consistent with the earlier observation that it was always selected as a predictor in the full models. FSGs that achieved particularly high balanced accuracies include KEGG B “metabolism of cofactors and vitamins” as well as KEGG B “amino acid metabolism”. All metrics generally achieved greater balanced accuracies at lower taxonomic levels, with genus delineations being the most accurately predicted (e.g. balanced accuracy ∼1.0 with AAI or 0.97 with FSG KEGG B “amino acid metabolism”), and phylum delineations being the least accurately predicted (balanced accuracy ∼0.9 with AAI or 0.8 with FSG KEGG B “metabolism of cofactors and vitamins”). The three gene categories whose FSGs generally achieved the highest balanced accuracy are KEGG B “metabolism of cofactors and vitamins”, KEGG B “amino acid metabolism”, and KEGG B “nucleotide metabolism”. This suggests that genes in these categories tend to play an important role in the differentiation of taxa, especially at lower taxonomic levels.

I point out that, since these balanced accuracies are achieved with FSGs within rarefied gene categories, they underestimate the balanced accuracies that could be achieved with FSGs within the full (i.e. non-rarefied) gene categories (Supplementary Fig. S5). For example, with rarefaction, the balanced accuracy achieved with FSG based on KEGG orthologs reaches 0.93 at genus level and 0.73 at phylum level (Fig. 3; Supplementary Data S7), whereas without rarefaction it reaches 0.99 at genus level and 0.84 at phylum level (Supplementary Fig. S5; Supplementary Data S8). Hence, for purposes of designing accurate classifiers, FSGs within non-rarefied gene categories should be used instead. Further, while some FSGs could be used as sole predictors for moderately to highly accurate classifiers (depending on taxonomic level), combining FSGs as predictors with AAI generally only has a small effect on balanced accuracy when compared with using AAI alone (Supplementary Table S5). Hence, a lot of the information contained in FSGs for delineating taxa is also contained in AAI; in other words, FSGs and AAI are to a substantial extent redundant with each other.

### Enumerating taxa and estimating global richness

To *de novo* cluster MAGs into taxonomic units and thus enumerate all marine prokaryotic taxa in the present data, I deployed clustering algorithms that use as input the pairwise classification probabilities between MAGs. Specifically, at each taxonomic level, I constructed an “affinity matrix” that specifies for each MAG pair the probability of being in the same taxon. For MAG pairs with *a priori* unknown classification (i.e. due to insufficiently precise taxonomic identities), the previously trained classifier was used to predict this probability, while for MAG pairs with known classification a probability of 0 or 1 was used, as appropriate, for the final taxon enumerations; however, the classifier was used for the cross-validation tests. I considered multiple well-established generic clustering algorithms, as well as two custom clustering algorithms constructed specifically for this study (“greedy groups” or GG, and “greedy groups refined” or GGR; pseudocode in Supplementary S1; python code in Supplementary Data S6). To my knowledge, algorithm GGR has not been published before. Following initial cross-validation tests, at each taxonomic level the algorithm with the best performance was used for the final clustering. At all taxonomic levels except families, GG was selected as the best performing algorithm, while at family level GGR was selected (Supplementary Table S6). Algorithm performance, measured in terms of the fraction of explained variance (*R*^2^) of taxon numbers across cross-validation rounds, was high in all cases, ranging from 0.968 at phylum level up to 0.999 at genus level. This demonstrates that *de novo* taxonomic clustering based on pairwise ingroup probabilities, in turn estimated based on a handful of genome similarity metrics, is a powerful alternative to conventional phylogenetic methods.

The *de novo* estimated number, of taxa in the data were 149 prokaryotic phyla, 337 classes, 978 orders, 2116 families, and 5679 genera (breakdown by domain in Supplementary Table S6). These numbers of course do not reflect the total richness of extant marine prokaryotic taxa; however, one may gain insight into the latter based on the distribution of taxa detected across surveys. Note that for purposes of assessing global taxon richness, one cannot rely solely on conventional taxonomic identifications of MAGs based on reference databases such as the GTDB [23], since the reference database would effectively define the “universe” of discoverable taxa; instead, a *de novo* taxon grouping, such as the one performed here, is necessary. To obtain a rough visual overview of the fraction of global taxonomic richness covered by this dataset, I computed taxon accumulation curves, separately at each taxonomic level (Fig. 4A–F). An accumulation curve, also known as a collector’s curve, shows for any given number of randomly chosen surveys the expected number of unique taxa that would be detected in those surveys [50]. An accumulation curve with substantially reduced slope at the maximum number of surveys, in other words approaching saturation, indicates that most extant taxa are already represented in the dataset. In particular, the ratio of the final slope of the curve over its initial slope, henceforth denoted *r*, corresponds to the fraction of taxa detected in a new study that are expected to be novel relative to this dataset (shown in Fig. 4A–F). At genus level, the accumulation curve has a slope ratio of *r* = 27%, which means that—on average—about 27% of prokaryotic genera detected in a new marine MAG survey are expected to be novel. On the other extreme, at phylum level, I found a slope ratio of only *r* = 2.1%, suggesting that on average only 2.1% of prokaryotic phyla detected in a new marine MAG survey would be novel, or put another way, 97.9% of detected phyla would already be covered by the present dataset. When accumulation curves are computed as a function of generated MAGs rather than as a function of the number of surveys, their saturation is even clearer (Supplementary Fig. S8). For example, at phylum level, the accumulation curve exhibits a slope ratio of only 0.13%, which means that each newly recovered MAG will only have a 0.13% chance of being in a novel phylum. This illustrates the increasing difficulty of discovering novel major prokaryotic clades in the ocean via genome-resolved metagenomics, either because most higher taxa have been discovered and/or because any remaining undiscovered taxa are rare.

The slope ratio *r* is only a rudimentary means to determine how many taxa remain undiscovered, because it does not account for the fact that some taxa may be rarer than others. To more precisely estimate the global number of marine prokaryotic taxa, I considered various statistical methods for estimating richness across surveys. To assess the robustness of estimates, I considered five different well-established methods, namely Chao2 [39], the improved Chao2 (iChao2; 40), incidence coverage-based estimator (ICE; 39), transformed weighted linear regression model (tWLRM; 41, 42), and breakaway [43]. All of these methods can account for differences in taxon prevalences, and breakway is particularly optimized for dealing with a high fraction of undetected diversity. Mathematically, each method is essentially based on the number of taxa that have been discovered in exactly one survey (*Q*_1_), the number of taxa discovered in exactly two surveys (*Q*_2_), and so on. Indeed, the statistically recommended way to estimate taxon richness is to model the incidence frequency counts *Q*_1_, *Q*_2_, …, in order to predict the number of unobserved taxa *Q*_0_ [39, 44, 51, 52]. Intuitively, widely abundant taxa with high detection probability contain very little information about undetected taxa, while rarely detected taxa (e.g. detected only once or twice) carry the most information about undetected taxa, and hence estimators typically rely on the low-frequency counts *Q*_1_, *Q*_2_, etc. [44]. Global taxon estimates for each taxonomic level and each estimation method are shown in Fig. 4. All methods generally yielded results within the same order of magnitude: the estimated global number of marine prokaryotic phyla ranged from 181 (method “ICE”) to 233 (“breakaway”), estimates for orders ranged from 1234 (“tWLRM”) to 1739 (“breakaway”), while estimates for genera ranged from 10 941 (“Chao2”) to 24 843 (“breakaway”). Thus, even if one were to consider the highest estimates, more than half of extant phyla, classes, and orders are already covered by the present dataset, while more than one-fifth of extant genera are covered by the dataset. In particular, recovering genomes that represent nearly all marine prokaryotic phyla and even genera appears to be a realistic target within reach. That said, I point out that estimating total richness from detected taxa is by its nature a difficult task [53], and it is possible that none of the models utilized above properly captures the abundance distribution of extremely rare taxa.

### Comparison with 16S similarities as a predictor

Prior to the widespread availability of whole-genome sequencing or MAGs, 16S rRNA gene sequences were a crucial tool for classifying prokaryotic taxa [13]. Unfortunately the 16S rRNA gene is typically poorly covered in MAGs due to difficulties in assembling this gene from metagenomes [14], which is why 16S sequences were omitted from the above analyses. To nevertheless examine how the predictive power of 16S rRNA gene sequence divergence compares with the genome-level predictors examined above, I also analyzed a dataset of 7314 marine-associated reference genomes with available full-length 16S sequence, obtained from the RefSeq database (details in Supplementary S2). Hence, pairwise 16S sequence similarities were considered as a possible predictor for classifier fitting, in addition to all similarity metrics discussed above for MAGs (data sizes in Supplementary Table S7, achieved classifier accuracies in Supplementary Table S8, backward sensitivity analyses in Supplementary Table S9). Based on the balanced classification accuracies achieved individually by each predictor (Supplementary Fig. S9), it becomes clear that 16S similarity is a relatively good predictor of taxonomic relationships particularly at the genus and class level (98% balanced accuracy). That said, multiple genome-level metrics outperformed 16S similarity, especially at the phylum level where 16S similarity only achieved a balanced accuracy of 83%. This may seem surprising, given that historically 16S had an important influence on the definition of many phyla. When considering the balanced accuracy averaged over all taxonomic levels (phylum to genus), AAI, FSGs based on all of KEGG, and FSGs based on metabolism genes all outperformed 16S as a sole predictor. When considering classifiers with arbitrarily selected predictor sets, 16S similarity was selected as a predictor at three out of five taxonomic levels (class, family, and genus). In two of those cases, 16S similarity was the predictor with the highest unique contribution to balanced accuracy, although it displayed high redundancy with other selected predictors (Supplementary Table S9).

It is worth noting that the achieved balanced accuracies for MAGs were generally comparable with—and in some cases even greater than—those achieved for RefSeq genomes (Table 1; Supplementary Table S8), despite the fact that 16S similarity was available as an additional predictor in the latter. The strongest difference was seen for phyla, where balanced accuracy increased from 0.926 to 0.974, and for classes, where balanced accuracy increased from 0.956 to 0.986, both of which constitute only moderate improvements. This suggests that the known quality issues of MAGs, compared with reference genomes, did not substantially compromise taxonomic delineation accuracy.

### Conclusions

This study introduced the use of neural network models for delineating marine prokaryotic taxa based on pairwise genome similarity metrics between MAGs, including AAI, ANI, and FSGs, within various gene categories. Classification accuracies were high at all taxonomic levels, but particularly so at lower levels such as genus or family, demonstrating that such methods can be powerful alternatives to traditional taxonomic classification approaches. Models constructed using individual metrics as sole predictors revealed that genes involved in the metabolism of cofactors and vitamins, genes involved in amino acid metabolism, and genes involved in nucleotide metabolism play a particularly important role in clade divergence at all levels. I stress, however, that classifier accuracy is only a proxy (more specifically, a lower bound) for information content, since in practice there will always be some residual information not properly captured by our imperfect models. It should also be kept in mind that historically prokaryotic taxonomic classification has been partly based on 16S sequence analyses [13], and hence classifier performances can be interpreted partly as correlations between various genome similarity metrics and 16S divergence.

This study also introduced the use of unsupervised clustering algorithms for reconstructing taxonomic relationships in a dataset *de novo*, i.e. without the use of a reference database or phylogenetic tree building. This opened the door to estimating global prokaryotic taxon richness in metagenomic datasets beyond the pool currently covered in reference databases. The computed taxon accumulation curves and statistical richness estimates both suggest that over half of extant marine prokaryotic phyla, classes, and orders, and over one-fifth of genera, are represented in the dataset analyzed here. The eventual recovery of MAGs from most of the ocean’s remaining prokaryotic genera thus appears within reach. As a word of caution, I emphasize that this conclusion only concerns the mere discovery of taxa, for example recovering at least one (high-quality) MAG within a novel genus. Each genus may in fact comprise a tremendous species and intraspecific diversity, and cataloguing and experimentally characterizing that diversity remains a much more formidable task.

## Supplementary Material

lqaf090_Supplemental_Files

## Data Availability

All data analyzed were published previously and obtained from public databases, as described in the Materials and methods. Data sources are listed in Supplementary Data S1. Details on all raw MAGs and genomes considered, including accession numbers, are available in Supplementary Data S2 and Data S4, respectively. Overviews of species genome bins are given in Supplementary Data S3 (MAGs) and Data S5 (RefSeq genomes). Python code implementing and demonstrating the greedy groups and greedy groups refined clustering algorithms is provided as Supplementary Data S6. All other software used in this paper have been described in the Materials and methods and are freely available online. Results from forward sensitivity analyses, including accuracies achieved individually by each similarity metric at each taxonomic level, are given in Supplementary Data S7 (FSGs based on rarefied gene groups) and Data S8 (non-rarefied gene groups).
