# Supplementary material for: Machine learning models for delineating marine microbial taxa
Source: NAR Genom Bioinform. 2025 Jun 19;7(2):lqaf090. doi: 10.1093/nargab/lqaf090 (PMC12204397; doi:10.1093/nargab/lqaf090)
Supplement: lqaf090_Supplemental_Files [file lqaf090_supplemental_files.zip › supplement.pdf]

Machine learning models for delineating marine microbial taxa - Supplementary Materials -

Stilianos Louca[1,2,*]

[1]Department of Biology, University of Oregon, USA
[2]Institute of Ecology and Evolution, University of Oregon, USA
[*]Corresponding author

S.1 Greedy clustering algorithms

In this section I provide pseudocodes for two unsupervised clustering algorithms, used to group MAGs into taxa based on estimated pairwise ingroup probabilities. These algorithms could in principle be used to cluster an arbitrary set of units (not necessarily MAGs) into groups based on previously determined pairwise ingroup probabilities. The first algorithm, "greedy groups" (GG), is a vanilla greedy clustering algorithm, for which no primacy is claimed and which is given here simply for clarity and completeness sake. The second algorithm, "greedy groups refined" (GGR), extends GG with an iterative group refinement process to obtain a more consistent grouping, i.e., where members of a group are all close to each other but distant to all other groups. The iterative refinements stop upon convergence or upon reaching a user-specified maximum number of allowed refinements. Both algorithms depend on a user-specified numeric parameter between 0 and 1, henceforth referred to as "affinity threshold" and denoted σ, which influences the propensity of units to be clustered together (a greater σ generally promotes smaller clusters). Both algorithms consider units in randomized order to break any unwanted correlations with unit labeling, and are thus non-deterministic.

To the best of my knowledge, GGR has not been published before. Tests revealed that, for the particular task of grouping MAGs into taxa, GG and GGR each outperformed common algorithms readily available in the `python` package `scikit-learn`, including DBSCAN, HDBSCAN and AffinityPropagation, for at least some of the considered taxonomic levels. In fact, at each taxonomic level the clustering algorithm ultimately selected was either GG or GGR (Table S6). Python code implementing both algorithms is provided as Data S6.

In the following, let N denote the number of units to be clustered (in our case, number of MAGs), and let $\mathbb{A}$ denote the $N \times N$ symmetric affinity matrix that lists pairwise ingroup probabilities for all unit pairs. Let the units be enumerated $1, .., N$, and at any time let $G_1, G_2, .. \subseteq \{1, .., N\}$ denote disjoint sets of units representing putative groups (clusters). In our case, N is the number of MAGs and $G_1, G_2, ..$ correspond to distinct taxa at a specific taxonomic level.

S.1.1 Algorithm "greedy groups"

Input: Number of units $N \in \mathbb{N}$, symmetric affinity matrix $\mathbb{A} \in [0, 1]^{N \times N}$ containing probabilities.

Input: Affinity threshold $\sigma \in (0,1]$. *# In this study values between 0.5 and 0.995 were considered*

Output: Groups $G_1, .., G_M \subseteq \{1, .., N\}$ such that $\bigcup_{i=1}^{M} G_i = \{1, .., N\}$.

$L \leftarrow \{\}$ *# L will eventually contain the desired groups*
Generate a random permutation $n_1, .., n_N$ of the indices $1, .., N$.
for $k = 1 : N$ **do**
  if $|L| = 0$ **then**
    Add $\{n_k\}$ to L *# Create a new group consisting only of unit n_k*
  else
    For each group $G \in L$, compute the mean affinity of n_k to G, i.e.:

$$a_G \leftarrow \frac{1}{|G|} \sum_{j \in G} \mathbb{A}_{n_k, j}$$

    Determine the group $G_o \in L$ with largest a_{G_o}
    if $a_{G_o} < \sigma$ **then**
      Add $\{n_k\}$ to L *# Create a new group consisting only of unit n_k*
    else
      Add n_k to G_o *# unit n_k is close to G_o, so add it*
    end if
  end if
end for

S.1.2 Algorithm "greedy groups refined"

Input: Number of units $N \geq 2$, symmetric affinity matrix $\mathbb{A} \in [0,1]^{N \times N}$ containing probabilities.
Input: Number of iterative refinements $R \in \mathbb{N}$. *# In this study $R = 100$ was used*
Input: Affinity threshold $\sigma \in (0,1]$. *# In this study values between 0.5 and 0.995 were considered*
Output: Groups $G_1, .., G_M \subseteq \{1, .., N\}$ such that $\bigcup_{i} G_i = \{1, .., N\}$.

Compute initial groups $G_1, G_2, .., G_M$ using greedy groups algorithm, with affinity threshold σ.
# Refine groups, by re-evaluating unit-to-group affinities using the current group configurations, and merging groups that appear close to each other
for $r = 1 : R$ **do**
  $\tilde{M} \leftarrow M$
  Define $\tilde{G}_1 \leftarrow \{\}, .., \tilde{G}_M \leftarrow \{\}$ *# these will become refined groups*
  Generate a random permutation $n_1, .., n_N$ of the indices $1, .., N$.
  for $k = 1 : N$ **do**
    For each $m \in \{1, .., M\}$ compute the mean affinity of n_k to G_m excluding n_k, i.e.:

$$a_m \leftarrow \frac{1}{|G_m \setminus \{n_k\}|} \sum_{j \in G_m \setminus \{n_k\}} \mathbb{A}_{n_k, j}$$

    For each $m \in \{M+1, .., \tilde{M}\}$ compute the mean affinity of n_k to $\tilde{G}_m$, i.e.:

$$a_m \leftarrow \frac{1}{\left|\tilde{G}_m\right|} \sum_{j \in G_m} \mathbb{A}_{n_k, j}$$

Determine $m \in \{1, .., \tilde{M}\}$ with the largest non-NaN a_m.
if $a_m < \sigma$ **then**
$\tilde{M} \leftarrow \tilde{M} + 1$, $\tilde{G}_{\tilde{M}} \leftarrow \{n_k\}$ *# Create a new group consisting only of unit n_k*
else
Add n_k to $\tilde{G}_m$
end if
end for
# replace old groups with new groups, omitting groups with no members left
$M \leftarrow \tilde{M}, G_1 \leftarrow \tilde{G}_1, .., G_M \leftarrow \tilde{G}_M$.
Remove any empty groups G_m, and update M and all group indices accordingly.
# merge some groups if needed, using an algorithm similar to greedy groups applied at group level
$\tilde{M} \leftarrow 1, \tilde{G}_1 \leftarrow G_1$ *# $\tilde{G}_1, .., \tilde{G}_{\tilde{M}}$ will become merged groups*
for $m = 2 : M$ **do**
For each $\tilde{m} \in \{1, .., \tilde{M}\}$, compute the mean affinity between G_m and $\tilde{G}_{\tilde{m}}$, i.e.:

$$a_{\tilde{m}} \leftarrow \frac{1}{|G_m| \cdot |\tilde{G}_{\tilde{m}}|} \sum_{k \in G_m} \sum_{j \in \tilde{G}_{\tilde{m}}} \mathbb{A}_{k,j}$$

Determine $\tilde{m} \in \{1, .., \tilde{M}\}$ with the largest $a_{\tilde{m}}$.
if $a_{\tilde{m}} < \sigma$ **then**
# none of the existing merged groups are close to group m, so create a new merged group
$\tilde{M} \leftarrow \tilde{M} + 1$
$\tilde{G}_{\tilde{M}} \leftarrow G_m$
else
Add all elements of G_m to $\tilde{G}_{\tilde{m}}$
end if
end for
Rename $M \leftarrow \tilde{M}, G_1 \leftarrow \tilde{G}_1, \ldots, G_M \leftarrow \tilde{G}_{\tilde{M}}$.
if $G_1, .., G_M$ are unchanged compared to iteration $i-1$ **then**
Halt clustering and return groups $G_1, .., G_M$.
end if
end for

S.2 Supplemental methods details

Genomes and 16S sequence divergence

In order to examine the performance of 16S rRNA gene sequence divergence as a predictor for taxonomic delineation, compared to the other predictors considered here, I also analyzed a set of reference genomes obtained from the NCBI RefSeq database. The reason for considering reference genomes rather than the MAGs is that 16S rRNA gene sequences are absent from the majority of MAGs due to difficulties with assembling that gene. Only genomes associated with the marine environment were considered. Genomes were downloaded on January 24, 2025 and were identified as marine either based on their geographic coordinates (if available), or based on their location metadata, specifically if they contained any of the keywords *marine*, *ocean*, *seafloor*,*seawater* in any of the following fields: *geo_loc*, *biosample_organism_name*, *metagenome_source*, *env_local_scale*, *isolation_source*, *isolation_site*, *ecosystem_type*, *habitat*. 16S rRNA gene sequences were extracted from each genome based on annotations provided by NCBI (file *rna_from_genomic.fna.gz*), keeping only the longest sequence per genome if multiple copies were found and only if that sequence was at least 1400 bp long. 16S sequences were thus retrieved for a total of 7314 marine-associated genomes; all subsequent analyses were restricted to this set of genomes. An overview of these genomes, including accession numbers, is available as Data S4. Taxonomic identities of genomes were taken from NCBI as-is. All other analyses of genomes, including clustering genomes to species genome bins (yielding 3175 bins), predicting and identifying protein-coding genes, calculating pairwise similarity metrics between genomes and model fitting, were performed similarly to MAGs to facilitate comparisons, with the exception that pairwise 16S rRNA sequence similarities (computed using `vsearch allpairs_global` with `-iddef 2`) were added to the pool of possible predictor variables. 16S similarities were computed using `vsearch allpairs_global` with `-iddef 2`, which computes similarity following pairwise alignment and according to (matching columns) / (alignment length - terminal gaps). Data sizes used for classifier selection and fitting are shown in Table S7. Achieved classifier accuracies for genomes are listed in Table S8. Backward sensitivities, i.e., measuring the contribution of each selected predictor to the overall classifier accuracy, are shown in Table S9.

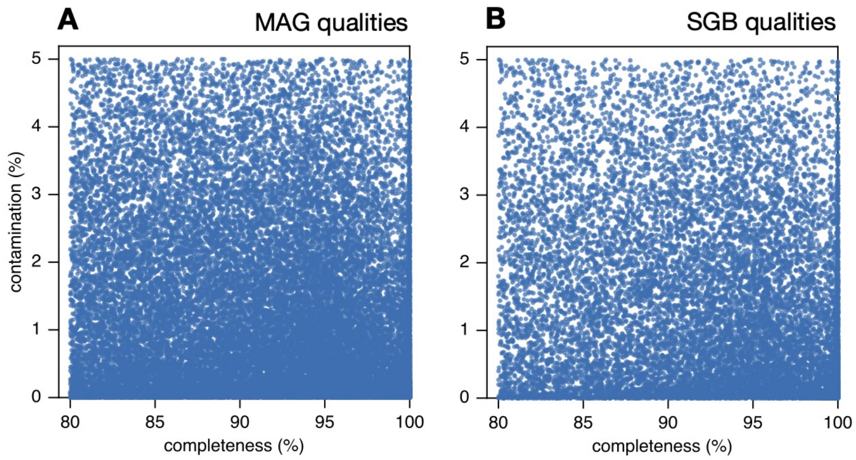

Figure S1: **MAG qualities.** (A) Completeness and contamination estimates for all raw MAGs, i.e. prior to binning into species genome bins. (B) Similar to A, but only showing species genome bin representatives.

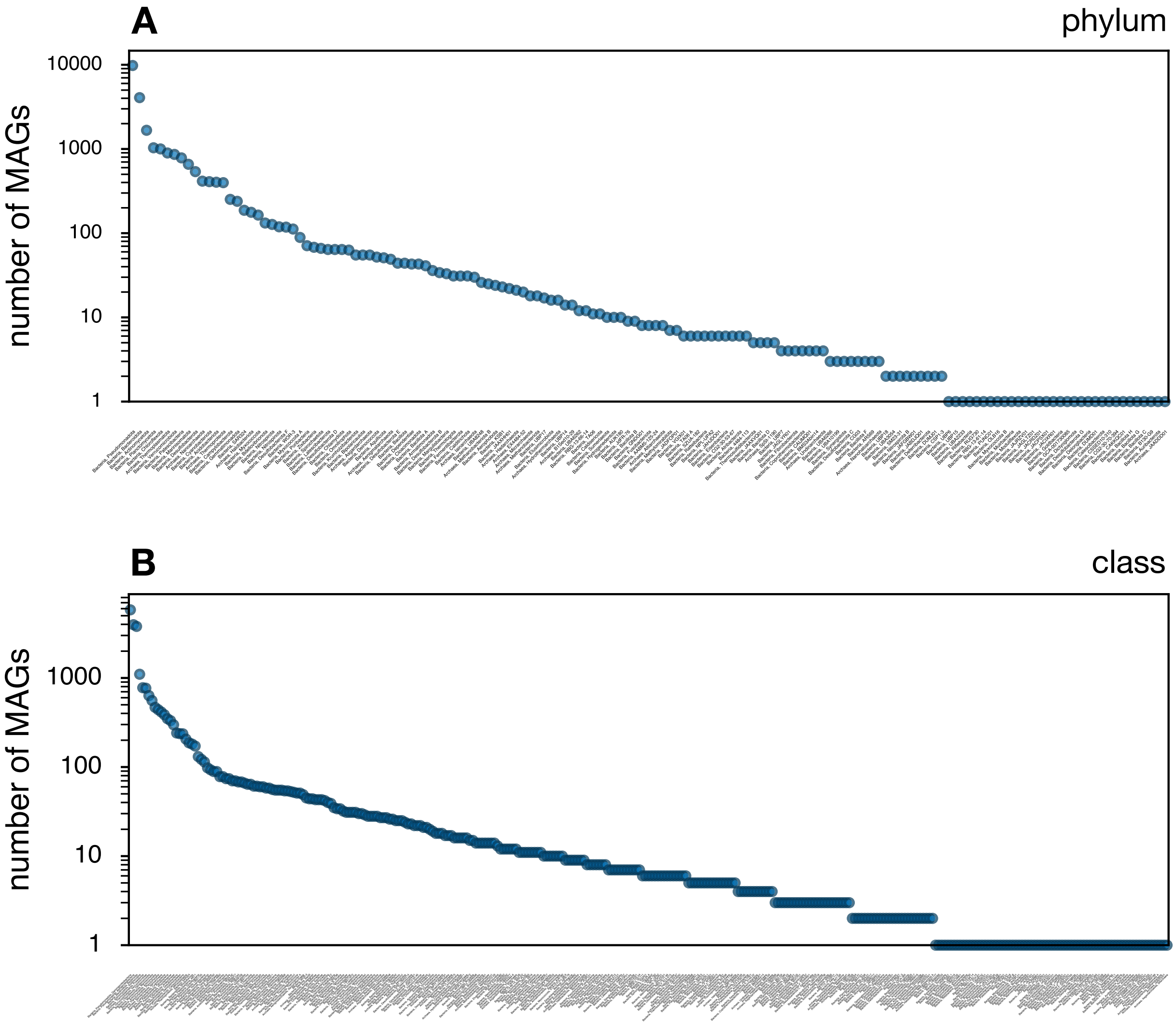

Figure S2: **Taxonomic representation in MAGs.** (A) Number of high-quality (completeness ≥80%, contamination ≤5%) MAGs associated with each named phylum. (B) Number of high-quality MAGs associated with each named class.

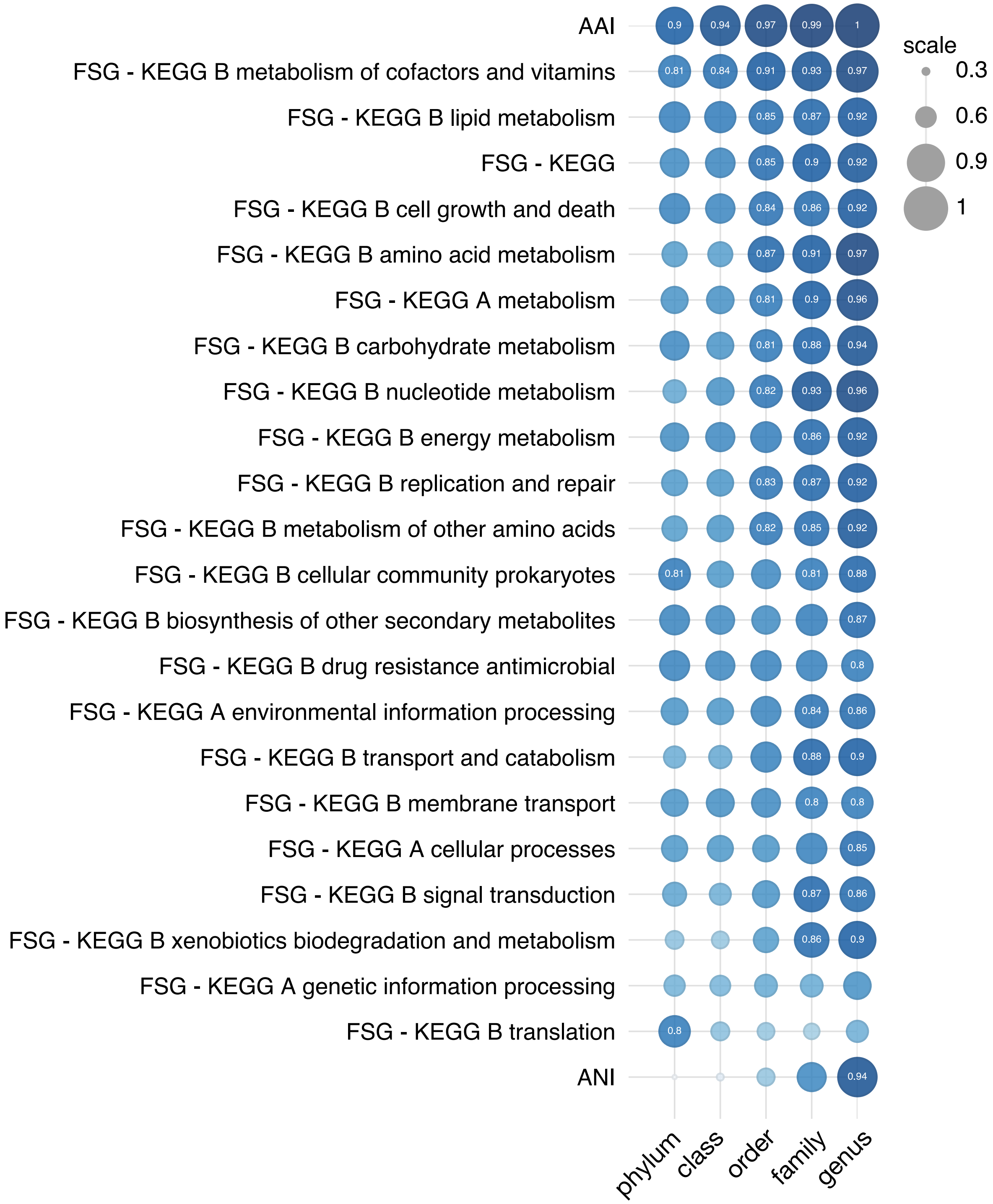

Figure S3: **True positive rates achieved by individual genome similarity metrics (rarefied gene sets, MAGs).** True positive rates ("sensitivities") achieved by classifier models based on individual metics of genomic similarity, including AAI (average aminoacid identity), ANI (average nucleotide identity) and FSGs (fractions of shared genes, restricted to various KEGG categories), separately for each taxonomic level. Larger and darker circles correspond to higher true positive rates. Values at or above 0.8 are written inside the circles. Metrics are listed in order of decreasing true positive rate (averaged over all taxonomic levels). Prior to computing FSGs, KEGG categories were rarefied to the same number of genes (115) per group, to avoid size-driven biases.

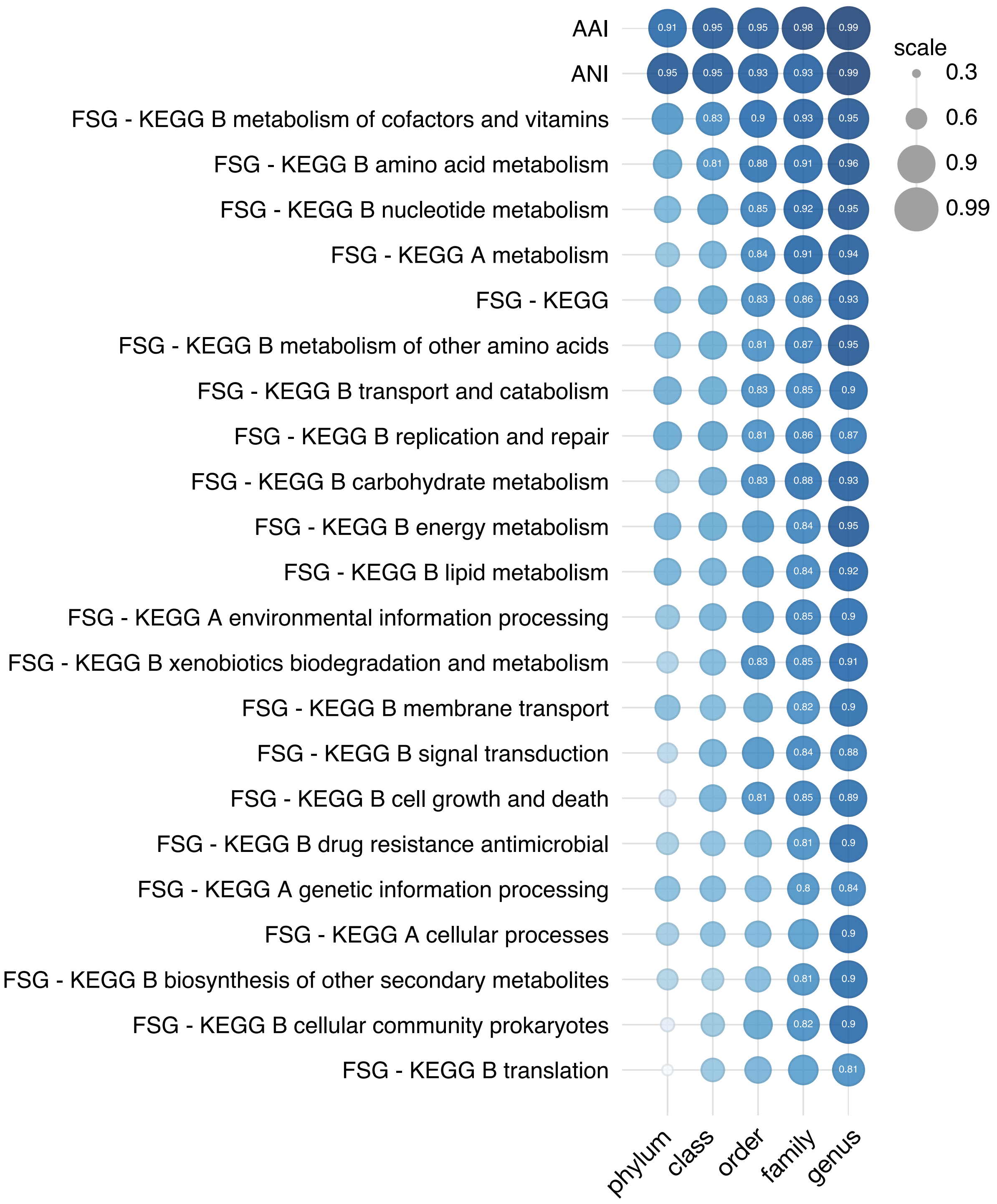

Figure S4: **True negative rates achieved by individual genome similarity metrics (rarefied gene sets, MAGs).** True negative rates ("specificities") achieved by classifier models based on individual metics of genomic similarity, including AAI (average aminoacid identity), ANI (average nucleotide identity) and FSGs (fractions of shared genes, restricted to various KEGG categories), separately for each taxonomic level. Larger and darker circles correspond to higher true negative rates. Values at or above 0.8 are written inside the circles. Metrics are listed in order of decreasing true negative rate (averaged over all taxonomic levels). Prior to computing FSGs, KEGG categories were rarefied to the same number of genes (115) per group, to avoid size-driven biases.

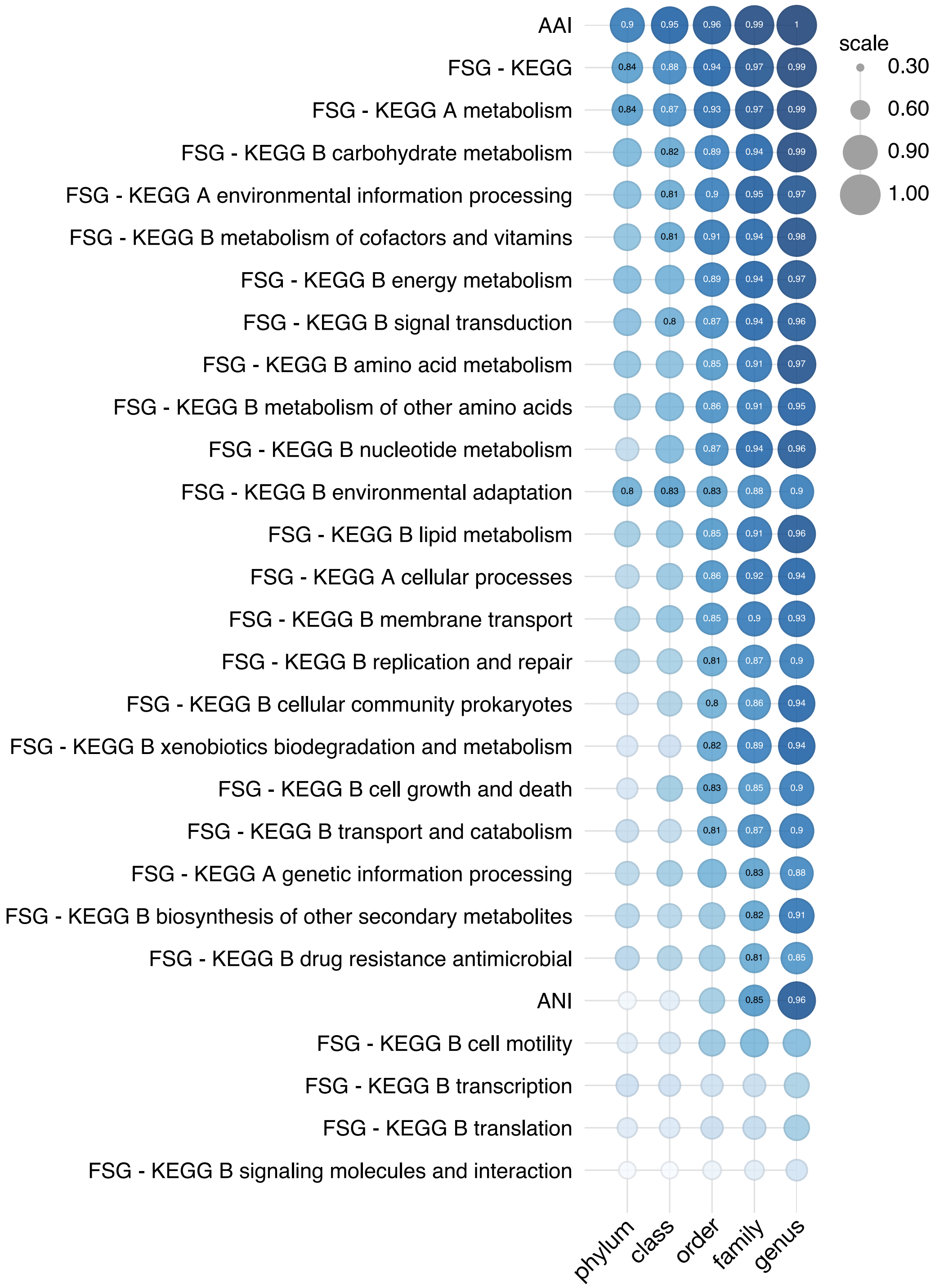

Figure S5: **Balanced accuracies achieved by individual similarity metrics (non-rarefied gene sets, MAGs).** Balanced classification accuracies achieved by models based on individual metics of genomic similarity, including AAI (average aminoacid identity), ANI (average nucleotide identity) and FSGs (fractions of shared genes, restricted to various KEGG groups), separately for each taxonomic level. Larger and darker circles correspond to higher accuracies. Balanced accuracies at or above 0.8 are written inside the circles. Metrics are listed in order of decreasing balanced accuracy (averaged over all taxonomic levels). For detailed values see Data S8. For balanced accuracies achieved with FSGs from gene sets rarefied to the same number of genes, see Fig. 3.

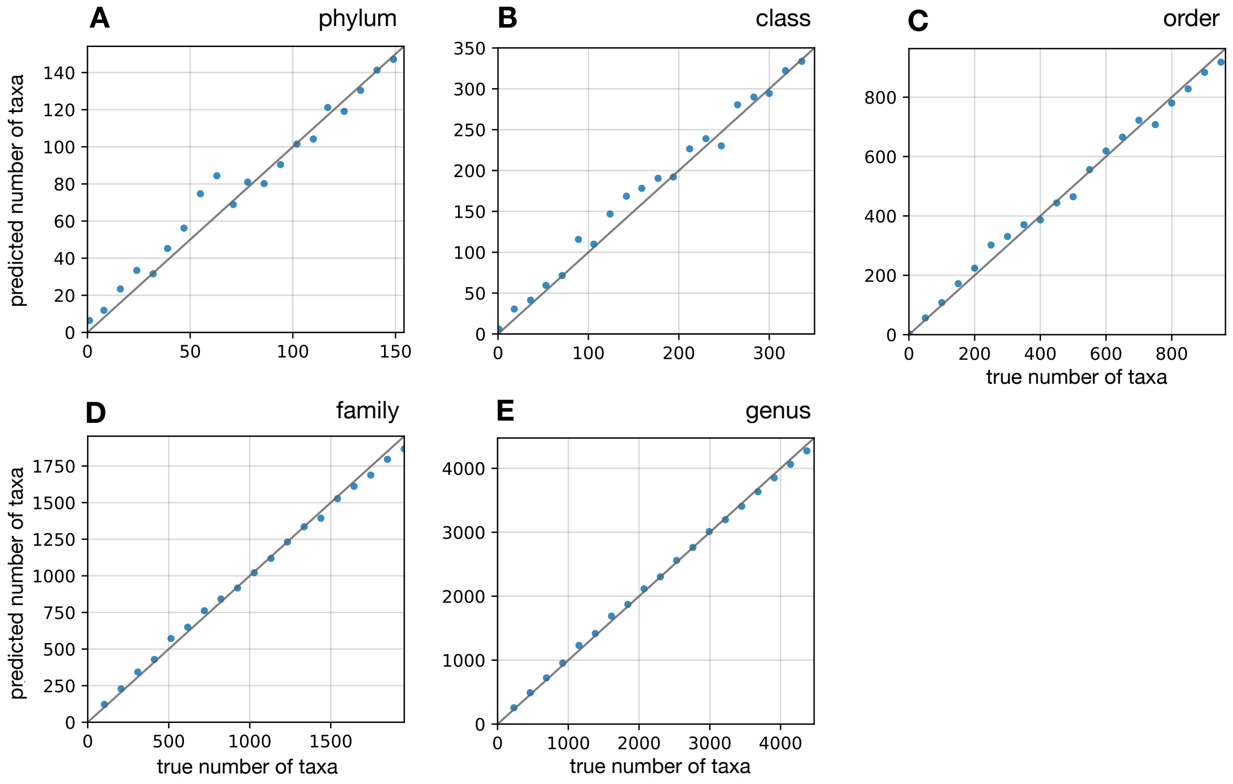

Figure S6: **Performance of taxon clustering algorithms (MAGs).** (A) Number of phyla predicted de novo by the selected clustering algorithm (vertical axis) based on predicted pairwise ingroup probabilities, for random subsets of MAGs of various sizes, compared to the true number of phyla in each MAG subset (horizontal axis). (B–E) Similar to A, but at lower taxonomic levels.

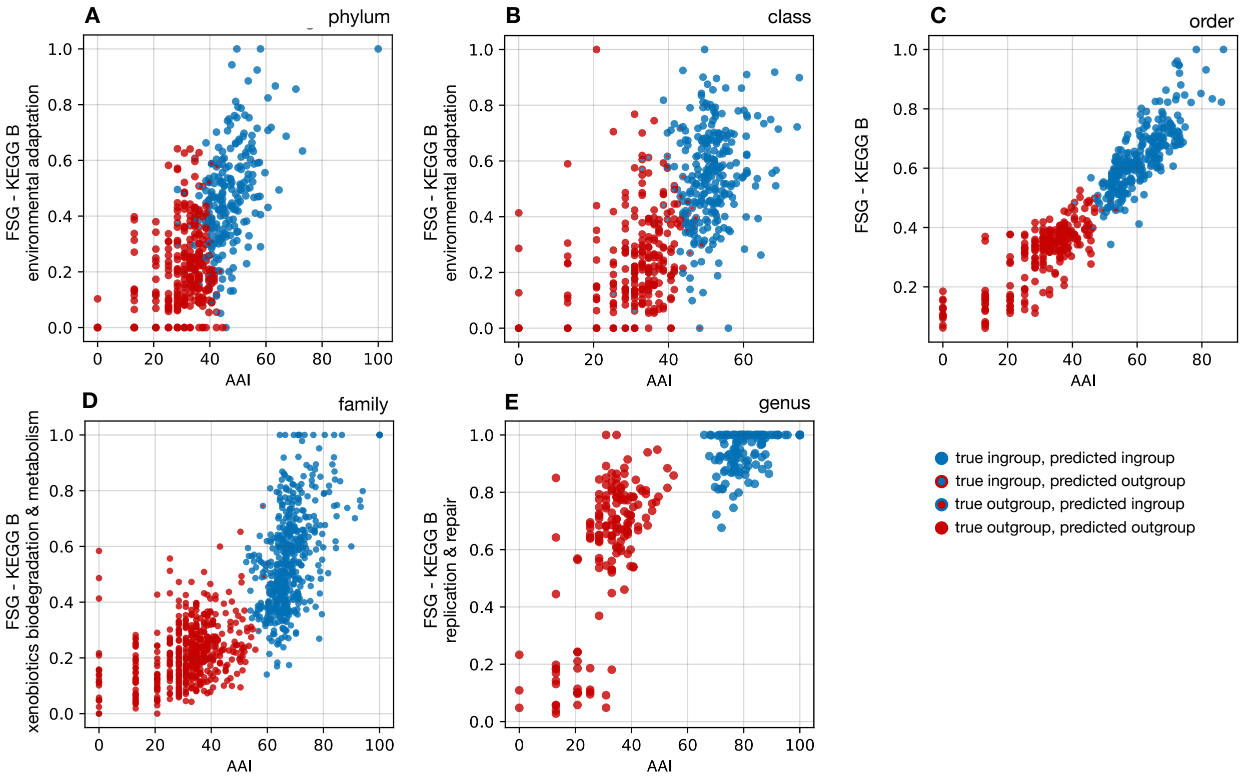

Figure S7: **Classifier test results (MAGs).** (A) Visual representation of classification test results of MAGs at phylum level. Each scatterpoint represents a sample in the test set, with the interior colored according to the true value (ingroup vs outgroup) and the perimeter colored according to the predicted value. Hence, single-color points represent correct predictions, while multi-color points represent erroneous predictions. Test samples have been balanced via rarefaction so that the numbers of true outgroup and true ingroup samples are equal, and further subsampled to 500 points, for visual readability. (B–E) Similar to (A), but at class, order, family and genus level. In (D) test samples are subsampled to 1000 points, and in (E) balancing reduced the number of displayed points down to 314.

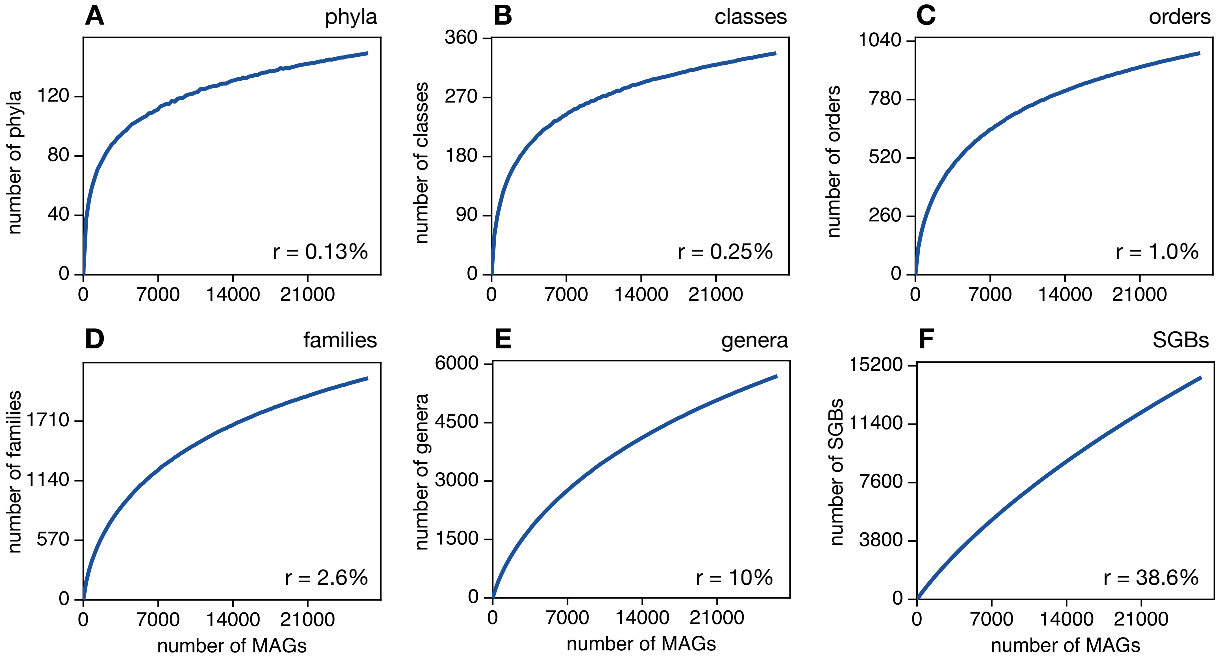

Figure S8: **Taxon accumulation curves, as MAGs are added.** (A) Accumulation curve of phyla over an increasing number of non-clustered MAGs. Every point in the curve shows the estimated number of phyla represented by a specific number of MAGs, averaged over multiple randomly chosen sets of MAGs. The number of represented phyla was estimated using our previously trained classifier and selected clustering algorithm, as described in the text. The ratio of the final (i.e., right-most) slope over the initial (i.e., left-most) slope, denoted r, is written in the figure; this slope corresponds to the probability of detecting a novel phylum when recovering a new MAG, i.e., a phylum not represented in the data analyzed here. (B–E) Similar to A, but for classes, orders, families and genera, respectively. (F) Accumulation curve of species genome bins over an increasing number of raw MAGs. For a similar figure showing accumulation curves over an increasing number of projects, see Fig. 4 in the main text.

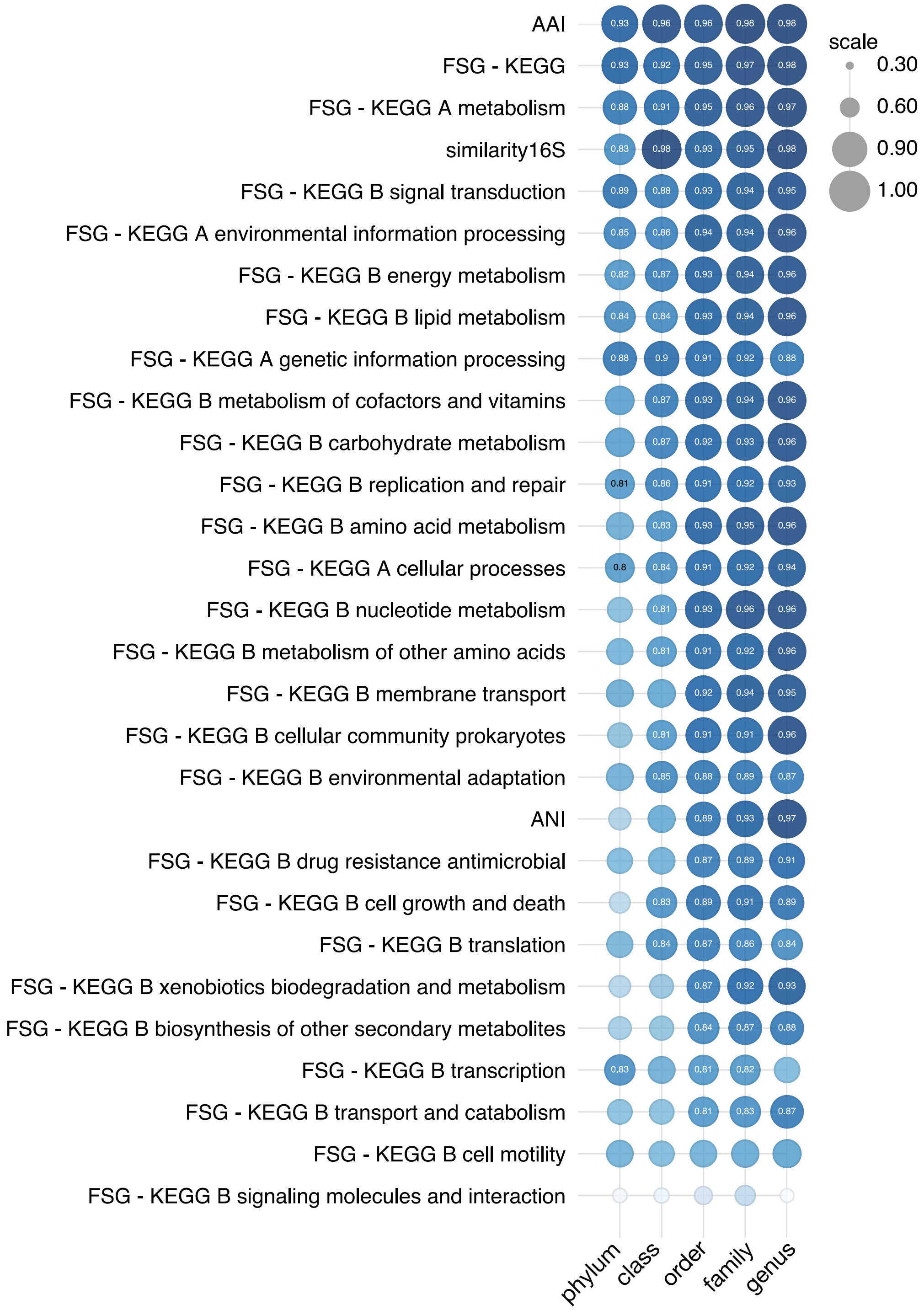

Figure S9: **Balanced accuracies achieved by individual similarity metrics (non-rarefied gene sets, RefSeq genomes).** Balanced classification accuracies achieved for RefSeq genomes by models based on individual metics of genomic similarity, including AAI (average aminoacid identity), ANI (average nucleotide identity) and FSGs (fractions of shared genes, restricted to various KEGG groups), separately for each taxonomic level. Larger and darker circles correspond to higher accuracies. Balanced accuracies at or above 0.8 are written inside the circles. Metrics are listed in order of decreasing balanced accuracy (averaged over all taxonomic levels).

Table S1: Overview of KEGG gene categories considered for predictor selection, including the number of genes found across MAGs or RefSeq genomes in each category. For each gene category, pairwise FSGs were computed between MAGs or genomes based on the presence/absence of those genes, or a rarefied subset of 100 of those genes (forward sensitivity analysis only). Note that gene categories with fewer than 100 genes were omitted from the forward sensitivity analyses with rarefaction.

**category**	**Ngenes (MAGs)**	**Ngenes (genomes)**
KEGG (all)	10256	10519
KEGG A cellular processes	722	739
KEGG A environmental information processing	1224	1199
KEGG A genetic information processing	563	512
KEGG A metabolism	3468	3639
KEGG B amino acid metabolism	629	624
KEGG B biosynthesis of other secondary metabolites	162	237
KEGG B carbohydrate metabolism	821	830
KEGG B cell growth and death	115	104
KEGG B cell motility	96	88
KEGG B cellular community prokaryotes	377	440
KEGG B drug resistance antimicrobial	124	169
KEGG B energy metabolism	656	644
KEGG B environmental adaptation	87	80
KEGG B lipid metabolism	260	242
KEGG B membrane transport	573	596
KEGG B metabolism of cofactors and vitamins	479	486
KEGG B metabolism of other amino acids	189	187
KEGG B nucleotide metabolism	210	206
KEGG B replication and repair	119	101
KEGG B signal transduction	642	604
KEGG B signaling molecules and interaction	50	33
KEGG B transcription	59	45
KEGG B translation	214	218
KEGG B transport and catabolism	143	114
KEGG B xenobiotics biodegradation and metabolism	327	347

Table S2: **Data sizes for machine learning (MAGs).** Overview of the number of data points (MAG pairs) in the training, validation and test datasets, used for classifiers at each taxonomic level. Also shown are the number of datapoints used to fit the final classifiers, i.e., after hyperparameter selection. Note that training sets were balanced via rarefaction to achieve equal representation of ingroup and outgroup MAG pairs, and subsequently subsampled to $\leq$ 20000 MAG pairs for computational efficiency. Data used for the final fit were also balanced via rarefaction.

tax. level	Ntraining	Nvalidation	Ntest	Nfinal
phylum	20 000	199 952	199 950	631 036
class	20 000	199 949	199 947	348 246
order	20 000	199 883	199 882	76 396
family	20 000	199 853	199 851	27 806
genus	2572	199 757	199 756	3 212

Table S3: **Selected hyperparameters (MAGs).** Overview of classifier hyperparameters selected at each taxonomic level, for MAGs.

tax. level	activation	alpha	hidden layer sizes
phylum	tanh	0.01	6, 6
class	tanh	0.01	6
order	tanh	0.01	3, 1
family	tanh	1	4, 4
genus	tanh	1	3

Table S4: **Classifier performances (MAGs, FSGs only).** Overview of achieved classification accuracies (true positive rate, true negative rate, balanced accuracy) and FSG metrics selected as predictors, at each taxonomic level. Only FSGs were considered as possible predictors.

tax. level	true pos.	true neg.	bal. acc.	predictors
phylum	0.891	0.902	0.897	FSG KEGG,<br>FSG KEGG A metabolism,<br>FSG KEGG A genetic information processing,<br>FSG KEGG B translation,<br>FSG KEGG B environmental adaptation,<br>FSG KEGG B transcription
class	0.914	0.916	0.915	FSG KEGG,<br>FSG KEGG A metabolism,<br>FSG KEGG B cell motility,<br>FSG KEGG B environmental adaptation
order	0.960	0.934	0.947	FSG KEGG,<br>FSG KEGG B environmental adaptation
family	0.977	0.966	0.971	FSG KEGG,<br>FSG KEGG B nucleotide metabolism
genus	0.994	0.986	0.990	FSG KEGG,<br>FSG KEGG B signaling molecules and interaction

Table S5: **Backward sensitivity analysis (MAGs).** Backward sensitivities of the classifiers, measured in terms of the reduction in balanced accuracy upon removal of a predictor and followed by new hyperparameter selection and training. Note that classifiers at different taxonomic levels may use different genome similarity metrics as predictors.

**predictor**	**phylum**	**class**	**order**	**family**	**genus**
AAI	0.0520	0.0912	0.0198	0.0959	0.0982
FSG KEGG	0.0042	-	0.0021	-	-
FSG KEGG B environmental adaptation	0.0122	0.0072	-	-	-
FSG KEGG B amino acid metabolism	0.0057	0.0040	-	-	-
FSG KEGG B xenobiotics biodegradation & metabolism	-	-	-	0.0037	-
FSG KEGG B replication & repair	-	-	-	-	0.00044

Table S6: **Selected clustering algorithms (MAGs).** Overview of clustering algorithms selected at each taxonomic level, their associated accuracies (measured in terms of the fraction of explained variance, R^2) and the total number of marine bacterial, archaeal and prokaryotic taxa predicted in the data. Also shown are the number of marine prokaryotic taxa predicted in the data using a slightly modified approach (denoted by *), whereby the taxonomic relationships (ingroup vs outgroup) between MAGs are predicted even if they are a priori known. Abbreviations: GG, greedy groups; GGR, greedy-groups-refined. The optimal affinity threshold, a parameter for the GG and GGR algorithms, is given in parentheses.

**tax. level**	**algorithm**	R^2	**bact. taxa**	**arch. taxa**	**prok. taxa**	**prok. taxa***
phylum	GG(0.6)	0.968	130	19	149	147
class	GG(0.6)	0.982	288	49	337	335
order	GG(0.9)	0.993	870	108	978	959
family	GGR(0.95)	0.996	1845	271	2116	2024
genus	GG(0.995)	0.999	5129	550	5679	5620

Table S7: **Data sizes for machine learning (RefSeq genomes).** Overview of the number of data points (RefSeq genome pairs) in the training, validation and test datasets, used for classifiers at each taxonomic level. Also shown are the number of datapoints used to fit the final classifiers, i.e., after hyperparameter selection. Note that training sets were balanced via rarefaction to achieve equal representation of ingroup and outgroup genome pairs, and subsequently subsampled to $\leq$ 20000 genome pairs for computational efficiency. Further, validation and test sets were subsampled to 200 000 samples for computational efficiency. Data used for the final fit were also balanced via rarefaction.

**tax. level**	**Ntraining**	**Nvalidation**	**Ntest**	**Nfinal**
phylum	20 000	200 000	200 000	1 196 104
class	20 000	200 000	200 000	608 586
order	20 000	200 000	200 000	181 540
family	20 000	200 000	200 000	106 852
genus	17 964	200 000	200 000	22 398

Table S8: **Classifier performances (RefSeq genomes).** Overview of achieved classification accuracies (true positive rate, true negative rate, balanced accuracy) and pairwise similarity metrics selected as predictors, at each taxonomic level. Note that in contrast to the MAG dataset, here pairwise 16S similarities were included as possible predictor.

tax. level	true pos.	true neg.	bal. acc.	predictors
phylum	0.974	0.974	0.974	AAI,<br>FSG KEGG,<br>FSG KEGG B energy metabolism,<br>FSG KEGG B transcription
class	0.988	0.983	0.986	AAI,<br>FSG KEGG,<br>16S similarity
order	0.972	0.955	0.963	AAI,<br>FSG KEGG B nucleotide metabolism
family	0.984	0.980	0.982	AAI,<br>16S similarity
genus	0.992	0.987	0.990	AAI,<br>FSG KEGG B metabolism of cofactors and vitamins,<br>16S similarity

Table S9: **Backward sensitivity analysis (RefSeq genomes).** Backward sensitivities of the classifiers, measured in terms of the reduction in balanced accuracy upon removal of a predictor and followed by new hyperparameter selection and training. Note that classifiers at different taxonomic levels may use different genome similarity metrics as predictors.

predictor	phylum	class	order	family	genus
AAI	0.012	0.0055	0.033	0.036	0.0014
FSG KEGG	0.0071	0.0026	-	-	-
FSG KEGG B energy metabolism	0.0055	-	-	-	-
FSG KEGG B transcription	0.016	-	-	-	-
16S similarity	-	0.021	-	0.0011	0.0091
FSG KEGG B nucleotide metabolism	-	-	0.0050	-	-
FSG KEGG B metabolism of cofactors & vitamins	-	-	-	-	0.00043
